# Pathogen distribution, antimicrobial resistance, and gut microbiota dysbiosis in patients with cirrhosis complicated by infection in northwestern China

**DOI:** 10.3389/fmicb.2026.1885174

**Published:** 2026-09-09

**Authors:** Hongjia Sun, Meimei Hu, Yanmei Xu, Yang Li, Shujuan An, Xiaorong Mao

**Affiliations:** 1 The First School of Clinical Medicine, Lanzhou University, Lanzhou, China; 2 Department of Medical Laboratory, The First Hospital of Lanzhou University, Lanzhou, China; 3 Department of Infectious Diseases, The First Hospital of Lanzhou University, Lanzhou, China

**Keywords:** antimicrobial resistance, butyrate, gut microbiota, infection, liver cirrhosis

## Abstract

**Introduction:**

Infection is a major complication of liver cirrhosis and is associated with substantial morbidity and mortality. However, regional data on pathogen distribution, antimicrobial resistance patterns, and gut microbiota and metabolic alterations among cirrhotic patients with infection in Northwest China remain limited. This study aimed to characterize these features and explore the associations between gut microbiota, short-chain fatty acids (SCFAs), and clinical inflammatory indices in cirrhotic patients with infection.

**Methods:**

We retrospectively analyzed 4,425 hospitalized patients with liver cirrhosis between January 2017 and October 2025, including 1,056 patients with concurrent infections, to characterize pathogen distribution and antimicrobial susceptibility patterns. In addition, a prospectively enrolled subgroup of 86 participants was analyzed for gut microbiota composition and fecal SCFA levels. Gut microbiota was characterized using 16S rRNA gene sequencing, and fecal SCFA concentrations were determined by appropriate analytical methods. The discriminatory ability of inflammatory indices, including the neutrophil-to-lymphocyte ratio (NLR), monocyte-to-lymphocyte ratio (MLR), and systemic immune-inflammation index (SII), was assessed using receiver operating characteristic (ROC) curve analysis.

**Results:**

The most frequently isolated Gram-negative bacteria were *Escherichia coli* (22.32%), *Klebsiella pneumoniae* (13.27%), *Acinetobacter baumannii* (7.36%), and *Pseudomonas aeruginosa* (5.07%), while the predominant Gram-positive bacteria were *Enterococcus faecium* (9.17%) and *Staphylococcus aureus* (6.39%). Fungi accounted for 4.58% of isolates. Antimicrobial susceptibility testing revealed substantial resistance to multiple antibiotics among the major pathogens. ROC analysis showed that NLR and MLR had high discriminative ability for distinguishing infected from non-infected cirrhotic patients, with AUCs of 0.9447 and 0.9083, respectively, while SII showed comparatively lower discriminative ability. In the microbiome subgroup, patients with spontaneous bacterial peritonitis (LCIF-SBP) exhibited distinct gut microbiota profiles compared with cirrhotic patients without infection (LCNOIF) and healthy controls. SCFA-producing genera, including *Bifidobacterium* and *Faecalibacterium*, were reduced, whereas *Escherichia-Shigella* was enriched. Fecal butyrate levels were significantly lower in the LCIF-SBP group than in the LCNOIF group. Correlation analysis showed that *Escherichia-Shigella* was negatively correlated with fecal butyrate levels and positively correlated with MLR.

**Discussion:**

Our findings provide regional data on pathogen distribution, antimicrobial resistance, gut microbiota alterations, and SCFA profiles among cirrhotic patients with infection in Northwest China. The observed associations between gut microbial dysbiosis, reduced butyrate levels, and inflammatory indices highlight potential links between intestinal microbial alterations and infection in cirrhosis. Further longitudinal, multicenter, and mechanistic studies are needed to determine whether these microbiota and metabolic alterations contribute causally to infection susceptibility and to evaluate their potential clinical relevance.

## Introduction

Cirrhosis is a severe chronic liver disease characterized by extensive fibrosis and nodular regeneration of hepatic tissue, which ultimately leads to liver failure (Ginès et al., 2021; Smith et al., 2019). In recent years, the global burden of cirrhosis has risen markedly, driven largely by the increasing prevalence of metabolic dysfunction-associated steatotic liver disease (MASLD) and alcoholic liver disease (Loomba et al., 2021; Gadi et al., 2024). In China, MASLD has emerged as one of the leading causes of cirrhosis, with a significantly rising prevalence, particularly among younger populations (Li et al., 2024; Man et al., 2023). Patients with liver cirrhosis are highly susceptible to infections due to impaired liver function, reduced immune capacity, and other contributing factors (Piano et al., 2024). The development of infections can further exacerbate liver damage, negatively impact patient prognosis and quality of life, and lead to increased healthcare costs and mortality rates (Piano et al., 2024; Bajaj et al., 2021). Bacterial infection represents a common form of co-infection among patients with liver cirrhosis, and the bacterial spectrum as well as antimicrobial resistance patterns may vary across different geographic regions (Mattos et al., 2020). Recent meta-analyses have provided critical insights into the evolving landscape of bacterial infections and antimicrobial resistance in cirrhosis. A 2025 global meta-analysis of 5,610 bacteremia cases reported a pooled bacteremia rate of 20%, with multidrug-resistant (MDR) infections accounting for 31% of cases and notable regional variation—both Asia and Europe exhibited MDR rates of approximately 35% (Paintsil et al., 2026). Another 2026 meta-analysis encompassing 169 studies and 35,180 infected patients further confirmed that Gram-positive bacteria are increasing over time and that MDR proportions are highest in low- and lower-middle-income countries and Southeast Asia (Cai et al., 2026). In Europe, a multicenter study from Italy reported that ESBL-producing *Enterobacteriaceae* accounted for over 30% of Gram-negative isolates in cirrhotic patients with spontaneous bacterial peritonitis (Piano et al., 2019), A Spanish cohort study of cirrhotic patients reported that MDR bacteria, especially ESBL-producing *Enterobacteriaceae*, accounted for up to 35% of nosocomial infections, making empirical third-generation cephalosporins essentially ineffective (Fernández et al., 2012). In Asia, a recent study from South Korea demonstrated that the proportion of carbapenem-resistant *Klebsiella pneumoniae* in cirrhotic patients with bacteremia increased from 8.1% to over 20% between 2010 and 2020 (Kim et al., 2024). In the Middle East, a study from Iran found that Gram-negative isolates from cirrhotic patients with spontaneous bacterial peritonitis exhibited high resistance rates to third-generation cephalosporins (Taherifard et al., 2021). These findings highlight the global threat posed by antimicrobial resistance to this vulnerable population, and it is necessary to formulate monitoring and management strategies for different regions in view of this geographical difference. Understanding the prevalent bacterial species and their resistance profiles in specific regions can assist clinicians in making evidence-based decisions regarding antibiotic selection, thereby enhancing treatment outcomes and mitigating the development of drug-resistant strains (Cannon et al., 2020).

The gut microbiota is essential in maintaining host immune homeostasis, nutrient metabolism, and intestinal barrier function (Smith et al., 2024). Patients with liver cirrhosis often experience intestinal flora imbalance due to impaired liver function, portal hypertension, and immune system dysfunction (Oh et al., 2020). This dysbiosis may further contribute to the development and progression of infections. Moreover, intestinal microbial metabolites, such as short-chain fatty acids (SCFAs), are involved in the regulation of inflammatory responses and immune functions. The presence of infection in cirrhotic patients may significantly alter the composition and activity of these metabolites (Lee and Suk, 2020).

Lanzhou is a major city in northwestern China, where environmental conditions, lifestyle, dietary habits, and other regional factors may influence the clinical characteristics of patients with liver cirrhosis complicated by infection. However, studies investigating pathogen distribution, antimicrobial resistance patterns, and alterations in gut microbiota and microbial metabolites in this population remain limited. Therefore, this study aimed to characterize the bacterial spectrum and antimicrobial resistance profiles of infections in patients with cirrhosis in Lanzhou, and to explore their potential associations with intestinal microbiota dysbiosis and metabolic changes. We analyzed demographic characteristics, laboratory parameters, pathogen types, and antibiotic susceptibility results. In addition, high throughput 16S rRNA gene sequencing was performed to assess the diversity and composition of the intestinal microbial community. Key microbial metabolites, including short-chain fatty acids, were also quantified. The findings of this study may provide a scientific basis for improving clinical diagnosis, optimizing treatment strategies, and evaluating prognosis in patients with liver cirrhosis complicated by infection, by clarifying the infection-associated microbial and metabolic alterations.

## Materials and methods

### Data collection

Inpatients with liver cirrhosis admitted to the First Hospital of Lanzhou University between January 2017 and October 2025 were selected as the study population. All patients were diagnosed with liver cirrhosis based on imaging examinations such as ultrasound and CT, as well as clinical manifestations or histopathological findings. The exclusion criteria were as follows: patients with incomplete medical records or who did not complete the entire course of diagnosis and treatment at our hospital; patients with severe comorbidities involving other major organs; and patients with a known history of malignancy. This study was approved by the Ethics Committee of the First Hospital of Lanzhou University. Patient data were retrospectively collected via the hospital information management system and included demographic information (age and gender), etiology of liver cirrhosis, presence of complications, comorbid conditions such as diabetes and hypertension, and whether invasive procedures were performed. Laboratory data were obtained through the laboratory information system and included results from biochemical analyses, complete blood counts, and preoperative coagulation profiles.

### Patient grouping

The baseline characteristics and laboratory data of the infected group (LCIF) and the non-infected group (LCNOIF) were compared and analyzed. Diagnostic criteria for infection in patients with liver cirrhosis were as follows: (1) Pulmonary infection: new pulmonary lesions accompanied by a body temperature exceeding 38 °C or below 36 °C, along with respiratory symptoms such as cough, sputum production, chest tightness, dyspnea, or chest pain, and a peripheral white blood cell count greater than 10.0 × 10^9^/L; (2) Spontaneous bacterial peritonitis (SBP): ascitic fluid polymorphonuclear cell count exceeding 250 cells/μL or a positive ascitic fluid bacterial culture, in the absence of any surgically treatable infectious source; (3) Bacteremia: positive blood culture obtained from a non-localized infection site; (4) Urinary tract infection: symptoms including frequency, urgency, dysuria, or flank pain, accompanied by a positive clean-catch urine culture or a urine routine showing white blood cell count greater than 15 cells per high-power field; (5) Skin and soft tissue infection: fever accompanied by signs of cellulitis; (6) Abdominal infection: including conditions such as cholangitis, pancreatitis, and diverticulitis; (7) Secondary peritonitis: endogenous peritoneal infection occurring in the context of ascitic fluid polymorphonuclear cell count exceeding 250 cells/μL or a positive ascitic fluid bacterial culture; (8) Bacterial enteritis: diarrhea and other gastrointestinal symptoms, confirmed by a positive stool culture.

### Isolation of bacterial pathogen

Clinical specimens submitted to the laboratory were processed in strict accordance with established standard operating procedures. Blood samples were incubated in the BacT/Alert3D 240 automated blood culture system (bioMerieux, France) at 37 °C for up to 5 days, with continuous automated monitoring for microbial growth, following the manufacturer's standard protocol. For other clinical specimens, including sputum, urine, and secretions, samples were inoculated onto appropriate culture media—such as blood agar, MacConkey agar, and chocolate agar—according to routine protocols, and subsequently incubated for 24 h at 37 °C. Following incubation, potential pathogenic bacteria were initially identified based on colony morphology, color, and growth characteristics, with representative colonies selected for further identification.

### Identification of bacterial pathogen

The identification of selected positive colonies was conducted using the VITEK MS microbial mass spectrometry identification system (bioMérieux, lEtoile, France). A suitable quantity of pure colonies was carefully extracted from the culture plate and uniformly distributed onto a mass spectrometry-specific target plate, followed by the application of a matrix solution. Once the matrix solution had dried, the target plate was inserted into the VITEK MS mass spectrometer. Bacterial species identification was subsequently determined based on the comparative analysis results.

### Antimicrobial sensitivity testing

The drug sensitivity test was conducted using the VITEK 2 automated system (bioMérieux, lEtoile, France). A pure bacterial colony was suspended in sterile saline to prepare a bacterial suspension of appropriate concentration. Once the turbidity of the suspension was adjusted to meet the required range, the suspension was transferred into a dedicated antimicrobial susceptibility testing card compatible with the VITEK 2 system. The card was then loaded into the VITEK 2 analyzer, which automatically performed incubation, detection, and data analysis. In certain specific cases, the Kirby-Bauer (K-B) disk diffusion method was employed as a supplementary method to confirm the antimicrobial susceptibility results. In this method, the standardized bacterial suspension was evenly spread onto Mueller-Hinton agar plates, followed by the placement of antibiotic-impregnated disks on the surface of the agar. The plates were incubated at 37 °C for 16 h, after which the diameters of the inhibition zones were measured. Bacterial susceptibility to various antibiotics was interpreted according to the Clinical and Laboratory Standards Institute (CLSI) 2022 guidelines (Clinical and Laboratory Standards Institute, 2022). All susceptibility testing results were originally recorded as minimum inhibitory concentrations (MICs) or inhibition zone diameters, and the CLSI 2022 breakpoints were applied uniformly to all isolates regardless of the year of collection (2017–2025), including those from 2025. This single unified interpretative standard was used consistently across all isolates to ensure data comparability over the entire study period, as applying different yearly breakpoints would render longitudinal resistance trend analysis unreliable. This approach is consistent with standard practices in retrospective antimicrobial resistance surveillance and is recommended by the WHO Global Antimicrobial Resistance System (GLASS) for data standardization. The detection of pathogenic bacteria in patients with liver cirrhosis complicated with infection from 2017 to 2025 was analyzed, including the distribution of positive specimens, the top six pathogens with detection rate and their drug sensitivity results.

Defined according to the international consensus criteria proposed by Magiorakos et al. (2012), MDR as non-susceptibility to ≥1 agent in ≥3 antimicrobial categories; XDR as non-susceptibility to ≥1 agent in all but ≤ 2 categories.

### Microbiological data deduplication and quality control

To ensure the validity of pathogen frequency and antimicrobial resistance analyses, the following standardized deduplication and quality control rules were applied to all culture-positive results:

(1) Patient-episode as the analytical unit: For patients with multiple positive cultures during the same hospitalization episode, only the first isolate of a given species was included in the frequency and resistance analyses; subsequent isolates of the same species from the same patient during the same episode were excluded, regardless of specimen type; (2) Polymicrobial infections: If a single patient had two or more distinct bacterial species isolated from the same or different specimens during the same episode (polymicrobial infection), each distinct species was counted separately as an independent isolate in the pathogen distribution analysis; (3) Repeated episodes: If a patient was readmitted with a new infection episode during the study period (2017–2025), each episode was treated as an independent event, and the first isolate from each new episode was included; (4) Exclusion of contaminants: Isolates considered as contaminants or colonizers—including coagulase-negative staphylococci (except *Staphylococcus lugdunensis*), *Corynebacterium* spp., *Bacillus* spp. (except *Bacillus anthracis*), *Micrococcus* spp., *Propionibacterium acnes*, and viridans group streptococci (when isolated from non-sterile sites)—were excluded unless they were: (a) recovered from blood cultures in ≥2 separate sets within 48 h, or (b) isolated from a normally sterile site (e.g., ascites, pleural fluid, and cerebrospinal fluid) with compatible clinical signs of infection; (5) Culture-negative cases: Patients with clinical diagnoses of infection but negative microbiological cultures (227/1,056, 21.5%) were not included in the pathogen distribution or antimicrobial resistance analyses but remained in the baseline clinical comparisons.

### Blood analysis and serology

All patients with cirrhosis underwent laboratory investigations to measure levels of Alanine Aminotransferase (ALT), Aspartate Aminotransferase (AST), Neutrophil (N), Lymphocyte (L), Monocyte (M), Platelet (PLT), Total Bilirubin (TBIL), Albumin (ALB), C-Reactive Protein (CRP). The data was extracted from patients' medical records. The derived ratios were calculated as follows: Neutrophil/lymphocyte ratio (NLR) = neutrophil count/lymphocyte count; monocyte-to-lymphocyte ratio (MLR) = monocyte count / lymphocyte count; SII = platelet count × neutrophil count/lymphocyte count; TBIL/ALB = total bilirubin/albumin; CRP/ALB = CRP/albumin.

### DNA extraction of patients gut microbiota and 16S rRNA sequencing

For the gut microbiota analysis, stool samples were prospectively collected from a subgroup of patients between January 2023 and December 2024. A total of 86 participants were enrolled, comprising 30 healthy controls (HC), 30 cirrhotic patients without infection (LCNOIF), and 26 cirrhotic patients diagnosed with spontaneous bacterial peritonitis (LCIF-SBP). The LCIF-SBP group was a subset of the overall infected cohort (LCIF) and was defined according to the diagnostic criteria for SBP described above. The microbiome methodology in this study was reported in accordance with the STORMS (Strengthening The Organization and Reporting of Microbiome Studies) checklist (Mirzayi et al., 2021), and is consistent with the methodology employed in a recently published study from our research group (An et al., 2025). To control for confounding factors known to influence gut microbiota composition, we applied strictly defined inclusion and exclusion criteria. For the microbiota subgroup (*n* = 86), participants were excluded if they met any of the following criteria: Exclusion criteria included: (1) Antibiotic or probiotic use within the past month; (2) Patients receiving enteral or parenteral nutrition, or those on special therapeutic diets or dietary supplements; (3) History of malignancy (other than well-controlled hepatocellular carcinoma); (4) Co-infection with HIV, CMV, or EBV; (5) Hematological diseases, solid organ transplantation, immunosuppressive therapy, or long-term corticosteroid use; (6) Use of enemas or non-prescribed laxatives/antidiarrheals within 2 weeks prior to stool collection; (7) Severe chronic kidney disease requiring dialysis, or severe cardiopulmonary disease.

Additionally, to prevent antibiotic-induced microbiota distortion, all fecal samples in the LCIF-SBP group were collected strictly before the administration of empirical antibiotic therapy, immediately after the diagnosis of infection was established. These rigorous criteria are the primary reason for the relatively modest sample size (*n* = 86), as the majority of cirrhotic patients admitted to our center had received prior therapy or had other conditions that could confound the analysis.

It is worth noting that for the gut microbiota analysis, we specifically selected patients with LCIF-SBP as the representative infected subgroup. This focused selection was based on the following rationale: (1) SBP is the most characteristic and prevalent infection in cirrhotic patients, and its pathogenesis is intimately linked to gut microbiota dysbiosis and bacterial translocation; (2) restricting the microbiome analysis to a single, well-defined infection type minimizes clinical heterogeneity and potential confounding introduced by different infection sources (e.g., pulmonary vs. urinary tract), thereby enhancing the interpretability of the microbial alterations. All fecal samples in the LCIF-SBP group were collected prior to initiation of empirical antibiotic therapy.

For all three groups, fecal samples were collected within 24 h of the index hospital admission (or, for healthy controls, at enrollment) and prior to the initiation of any pharmacological intervention during that admission, including empirical antibiotic therapy, diuretics, or other newly instituted medications; sampling therefore preceded, rather than followed, the clinical course and total length of stay of the index hospitalization. Fresh fecal samples were collected using sterile containers, immediately placed on ice, transported to the laboratory within 2 h, aliquoted into cryovials, and stored at −80 °C until further analysis. For patients with infection, fecal samples were collected before initiation of empirical antibiotic therapy. Total bacterial genomic DNA was extracted using the cetyltrimethylammonium bromide (CTAB) method. DNA integrity was confirmed by 1% (w/v) agarose gel electrophoresis run at 80 V for 40 mins, with genomic DNA appearing as a single high-molecular-weight band without detectable smearing or degradation. DNA concentration and purity were assessed using a NanoDrop 2000 spectrophotometer (Thermo Fisher Scientific, Waltham, MA, USA). Only samples with A260/A280 ratios between 1.8 and 2.0 and A260/A230 ratios ≥2.0, and concentrations ≥20 ng/μL, were used for subsequent 16S rRNA gene amplification.

The V3–V4 hypervariable region of the 16S ribosomal RNA gene was amplified using the primer pair 341F (5′-CCTACGGGNGGCWGCAG3′) and 805R 5′-GACTACHVGGGTATCTAATCC3′). PCR amplification was performed in a total volume of 25 μL containing 25 ng of template DNA, 12.5 μL of 2× Phusion Hot Start Flex Master Mix, and 2.5 μL of each primer (10 μM). Thermocycling conditions were: initial denaturation at 98 °C for 30 s; 32 cycles of denaturation at 98 °C for 10 s, annealing at 54 °C for 30 s, and extension at 72 °C for 45 s; followed by a final extension at 72 °C for 10 min. The resulting amplicons were purified and recovered using AMPure XT beads (Beckman Coulter Genomics, Danvers, MA, USA), and their concentration was quantified with a Qubit 3.0 fluorometer (Invitrogen, USA). The amplicon library fragment size was validated using an Agilent 2100 Bioanalyzer (Agilent, USA), as described by Walker et al. (2023), in conjunction with the Library Quantification Kit (Kapa Biosciences, Woburn, MA, USA). Sequencing was performed on the Illumina NovaSeq 6000 platform with paired-end 250 bp reads (PE250). Raw sequencing data were processed through the following bioinformatics pipeline: (a) primer and adapter sequences were removed using Cutadapt v1.9; (b) paired-end reads were merged based on overlapping regions using FLASH v1.2.8; (c) quality filtering was performed using fqtrim v0.94, with a sliding window of 100 bp and a minimum average quality score of 20; reads shorter than 100 bp after trimming or containing >5% ambiguous bases (N) were discarded; (d) chimeric sequences were identified and removed using Vsearch v2.3.4. Denoised amplicon sequence variants (ASVs) were generated using the DADA2 (Divisive Amplicon Denoising Algorithm 2) algorithm within the QIIME2 framework. ASVs represent exact biological sequences resolved to single-nucleotide differences, providing higher taxonomic resolution and improved reproducibility compared to traditional operational taxonomic unit (OTU) clustering at 97% similarity. Singleton ASVs were removed. Taxonomic classification was performed against the SILVA v138 reference database. It should be noted that 16S rRNA gene sequencing of the V3–V4 region provides taxonomic resolution primarily at the genus level; species-level assignments should be interpreted with caution, as closely related species within the same genus may share identical or nearly identical V3–V4 sequences.

### Metabolomic study of stool samples

Collect the stool sample in 2 mL EP tubes and extract with 1 mL of pure water, vortexing for 10 s. Homogenize using a ball mill for 4 mins at 40 Hz, followed by 5 mins of ultrasound treatment in ice water; repeat this process three times. Centrifuge at 2,000 × g and 4 °C for 20 mins. Transfer 0.8 mL of the supernatant to a new 2 mL EP tube, add 0.1 mL of 50% H2SO4 and 0.8 mL of an extracting solution (25 mg/L in methyl tert-butyl ether) as an internal standard, and vortex for 10 s. Perform oscillations for 10 mins and ultrasound treatment for another 10 mins in ice water. Centrifuge at 8,000 × g and 4 °C for 15 mins. Store at −20 °C for 30 mins, then transfer the supernatant to a fresh 2 mL glass vial for gas chromatography-mass spectrometry (GC-MS) analysis.

GC-MS Analysis was performed using a SHIMADZU GC2030-QP2020 NX system (Kyoto, Japan) with an HP-FFAP capillary column (Agilent, California, USA). A 1 μL sample was injected in a 5:1 split mode. Helium served as carrier gas, with a front inlet purge flow of 3 mL/min and a column flow rate of 1.2 mL/min. The temperature program started at 50 °C for 1 min, increased to 150 °C at 50 °C/min for 1 min, then to 170 °C at 10 °C/min, to 225 °C at 25 °C/min for 1 min, and finally to 240 °C at 40 °C/min for 1 min. Injection, transfer line, quad, and ion source temperatures were set at 240 °C, 240 °C, 150 °C, and 240 °C, respectively. The system operated in electron impact mode at −70 eV, acquiring mass spectrometry data in Scan/SIM mode with an m/z range of 33–150 after a 3.75 min solvent delay.

### Statistical analysis

Microbial identification and antimicrobial susceptibility data were analyzed utilizing Microsoft Excel and WHONET 5.6, whereas the remaining datasets were processed using SPSS version 22.0. Normally distributed continuous variables were presented as mean ± standard deviation (SD), while non-normally distributed data were presented as the median and interquartile range (IQR) and were analyzed using the Mann–Whitney *U* test. For the comparison of quantitative variables between two groups, an unpaired *t*-test was employed when the data conformed to a normal distribution and demonstrated homogeneity of variance; otherwise, the Mann–Whitney *U* test was utilized. Categorical data were represented as absolute numbers (*n*, indicating the number of isolates) and percentages (%), with the chi-square (χ^2^) test employed for statistical analysis. The accuracy of infection discrimination was assessed using exploratory receiver operating characteristic (ROC) curve analysis, with a 95% confidence interval (95% CI) and the area under the curve (AUC). Spearman correlation analysis was performed to assess the relationships among gut microbiota composition, fecal short-chain fatty acid levels, and laboratory inflammatory indices. It should be noted that some of these laboratory parameters were also considered in the composite clinical assessment of infection, which may introduce incorporation bias and lead to overestimation of their discriminatory performance. Therefore, the ROC results should be interpreted as exploratory assessments of discriminatory capacity rather than as validation of independent diagnostic biomarkers. Notably, NLR, MLR, and SII are derived composite indices not directly included in infection diagnostic criteria, which may partially mitigate this concern for these specific parameters. Alpha diversity was assessed using the Chao1 index (species richness) and Shannon index (species evenness), calculated at the ASV level. Differences in alpha diversity between groups were tested using the non-parametric Kruskal–Wallis test followed by pairwise Mann–Whitney *U* tests. Beta diversity was evaluated using Bray-Curtis dissimilarity metrics, visualized by PCoA, and statistically assessed using PERMANOVA (permutational multivariate analysis of variance) with 999 permutations. Differential taxa were identified using the linear discriminant analysis effect size (LEfSe) method with default parameters (LDA score threshold > 2.0, *P* < 0.05). Spearman rank correlation analysis was used to assess associations between microbial genera, SCFA levels, and clinical parameters. All significance tests were two-sided, and a *P* < 0.05 was considered indicative of statistical significance.

### Ethics statements

This study was conducted in accordance with the Declaration of Helsinki and the ethical guidelines for biomedical research involving human subjects in China. The study protocol was approved by the Ethics Committee of the First Hospital of Lanzhou University (Approval number: LDYYLL2022-24). For the retrospective analysis of clinical data and bacterial isolates, the Ethics Committee granted a waiver of written informed consent in accordance with Article 32 of the Measures for Ethical Review of Life Sciences and Medical Research Involving Humans (2023, China), as the research involved anonymized data with no additional risk to participants. For the prospectively collected fecal samples from the microbiota subgroup (*n* = 86), all participants provided written informed consent prior to enrollment.

## Results

### Socio-demographic characteristics

**Figure 1:**
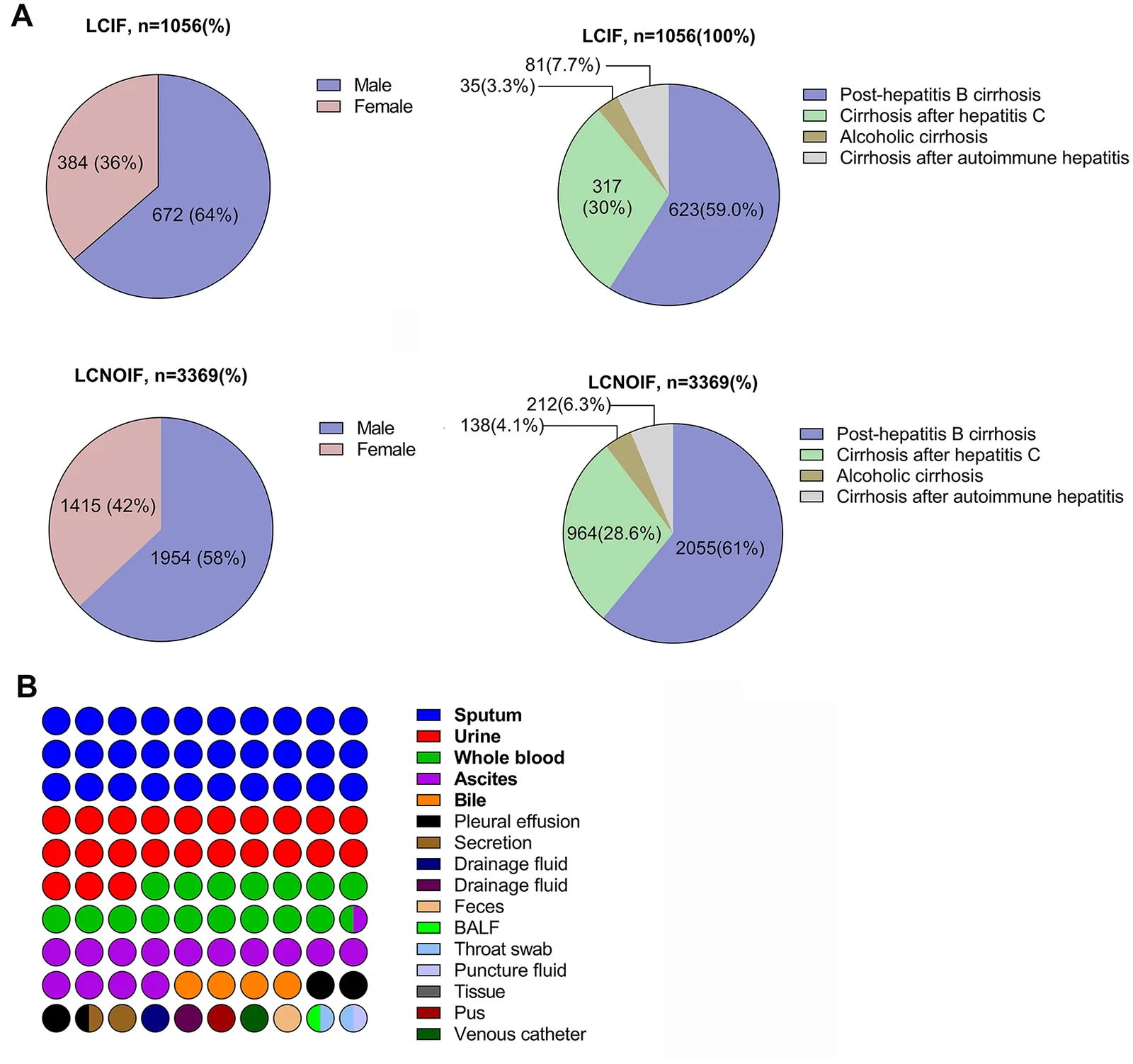
Baseline characteristics and specimen distribution in cirrhotic patients with and without infection. **(A)** Comparison of demographic and etiological characteristics between cirrhotic patients with infection (LCIF) and those without infection (LCNOIF). **(B)** Distribution of clinical specimen types from which pathogens were isolated in the LCIF group. Percentages were calculated based on the total number of non-duplicate isolates (*n* = 829).

A total of 4,425 patients with liver cirrhosis who were admitted to our hospital between January 2017 and October 2025 were enrolled in the First Hospital of Lanzhou University. The infected group comprised 1,056 cases (672 males and 384 females), with a mean age of 51.42 ± 10.50 years. The non-infected group included 3,369 cases (1,954 males and 1,415 females), with a mean age of 53.46 ± 11.58 years. Given the large sample size, statistically significant differences were observed in age (*P* < 0.001) and sex distribution (*P* = 0.001) between the two groups. No statistically significant differences were observed regarding the etiology of liver cirrhosis (viral, alcoholic, autoimmune, or other liver diseases; Figure 1A).

### Laboratory test values

In our case, laboratory test values showed significant differences between cirrhotic patients with infection (LCIF) and without infection (LCNOIF). NLR, MLR, SII, TBIL/ALB, CRP/ALB in LCIF patients was significantly higher than that of LCNOIF patients (Table 1). Meanwhile, ALT/AST was lower in LCIF patients than in LCNOIF patients, although the difference was not significant (Table 1).

**Table 1:** Descriptive statistics of laboratory indicators in patients with post-hepatitis B cirrhosis complicated with infection and non-infected cirrhosis in Lanzhou from 2017 to 2025.

Laboratory parameters	LCIF (*n* = 1,056) *M* (*Q*1, *Q*3)	LCNOIF (*n* = 3,369) *M* (*Q*1, *Q*3)	*P-v*alue
Neutrophil/lymphocyte ratio (NLR)	15.05 (6.60, 18.85)	2.49 (1.00, 6.30)	<0.0001
Monocyte/lymphocyte ratio (MLR)	1.88 (0.89, 2.41)	0.25 (0.12, 0.35)	<0.0001
Systemic Immune-Inflammation Index (SII)	817.30 (272.79, 1052.12)	147.70(32.44, 544.32)	<0.0001
Alanine Aminotransferase (ALT)/Aspartate Aminotransferase (AST)	1.26 (0.32, 5.06)	1.46 (0.36, 6.00)	0.0593
Total Bilirubin (TBIL)/Albumin (ALB, μmol/g)	3.31(0.95, 11.05)	1.82(0.61, 4.84)	<0.0001
CRP (C-Reactive Protein, mg)/Albumin (ALB, g)	0.79 (0.27, 1.14)	0.30 (0.14, 0.43)	<0.0001

SII = (Neutrophil × Platelet)/lymphocyte.

**Figure 2:**
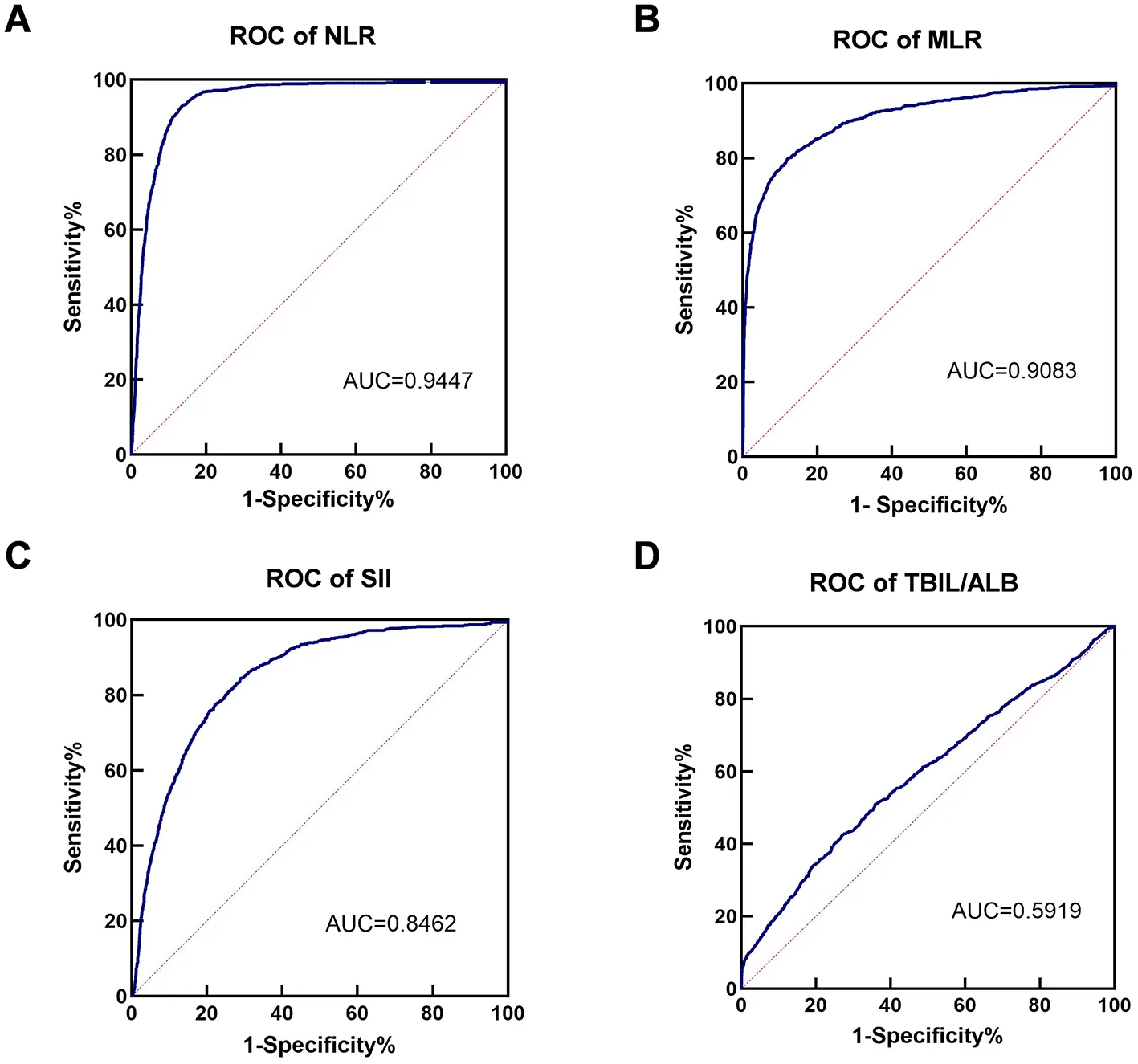
**(A–D)** Receiver operating characteristic (ROC) curves of four laboratory indicators (NLR, MLR, SII, and TBIL/ALB) for discriminating cirrhotic patients with infection (LCIF) from those without infection (LCNOIF). ROC curves for four parameters: NLR, MLR, SII, and TBIL/ALB. The AUC values are shown in each panel.

To further evaluate the exploratory discriminatory capacity of these indicators in identifying infection among cirrhotic patients, receiver operating characteristic (ROC) curve analysis was performed for four parameters (Figure 2). Among them, NLR (AUC = 0.9447) and MLR (AUC = 0.9083), both of which also exhibited excellent discriminatory ability between the LCIF and LCNOIF groups. SII showed good supplementary discriminatory capacity, with an AUC value of 0.8462, suggesting that this composite inflammatory index contributes meaningfully to infection identification in this patient population. In contrast, TBIL/ALB yielded a substantially lower AUC of 0.5919, with its ROC curve approaching the reference diagonal, indicating limited standalone diagnostic value for distinguishing infected from non-infected cirrhotic patients.

Critically, it should be noted that WBC and CRP/ALB, two parameters initially considered for this analysis, are components of the composite clinical criteria used to define infection. Because their inclusion would introduce an inherent “incorporation bias” into the ROC analysis, potentially resulting in overestimation of the observed AUC values, these two parameters were excluded from the ROC analysis presented here. Consequently, the ROC results presented in this study should be interpreted strictly as exploratory assessments of discriminatory capacity rather than as validation of independent diagnostic biomarkers. Specifically, NLR, MLR, and SII are derived composite indices not directly included in the infection diagnostic criteria, which may partially mitigate this concern for these specific parameters. Nevertheless, these non-invasive and routinely available indices may serve as adjunctive tools to support the early clinical suspicion of infection in cirrhotic patients, facilitating timely therapeutic decision-making.

### Detection of pathogenic bacteria in patients with liver cirrhosis complicated by infection

Among the 1,056 patients with clinically diagnosed infections, 829 patients (78.5%) had at least one positive culture episode. After applying the standardized deduplication rules described in the Section Materials and methods, a total of 829 non-duplicate isolates derived from 829 distinct patient-episodes were included in the final pathogen distribution and antimicrobial resistance analyses (Table 2). The remaining 227 patients (21.5%) were clinically diagnosed with infections based on established criteria but had negative culture results; these patients were excluded from the microbiological analyses but remained in the baseline comparisons. Among all clinical specimen types, the most frequently identified pathogens were derived from sputum, urine, whole blood, ascites, and bile, as illustrated in Figure 1B. Among these 829 non-duplicate isolates, Gram-negative bacteria were mainly *Escherichia coli* (185 isolates, 22.32%), *Klebsiella pneumoniae* (110 isolates, 13.27%), *Acinetobacter baumannii* (61 isolates, 7.36%), and *Pseudomonas aeruginosa* (42 isolates, 5.07%). Gram-positive bacteria were mainly *Enterococcus faecium* (76 isolates, 9.17%) and *Staphylococcus aureus* (53 isolates, 6.39%). Additionally, 38 fungal isolates (4.58%) were identified, including *Candida albicans, Candida tropicalis, Candida parapsilosis*, and *Aspergillus fumigatus*. Detailed data are presented in Table 2.

**Table 2:** Detection of pathogenic bacteria in patients with liver cirrhosis complicated with infection from 2017 to 2025.

Species of pathogenic bacteria	Number of isolates	Proportion (%)
*Escherichia coli*	185	22.32%
*Klebsiella pneumoniae*	110	13.27%
*Enterococcus faecium*	76	9.17%
*Acinetobacter baumannii*	61	7.36%
*Staphylococcus aureus*	53	6.39%
*Pseudomonas aeruginosa*	42	5.07%
*Enterobacter cloacae*	35	4.22%
*Stenotrophomonas maltophilia*	29	3.50%
*Klebsiella oxytoca*	26	3.14%
*Enterococcus faecalis*	23	2.77%
*Acinetobacter Johnson*	16	1.93%
*Pseudomonas fluorescens*	12	1.45%
*Streptococcus anginosus*	12	1.45%
*Staphylococcus epidermidis*	10	1.21%
*Haemophilus influenzae*	9	1.09%
*Salmonella Kleinfeld*	8	0.97%
*Citrobacter freundii*	8	0.97%
*Burkholderia cepacia*	6	0.72%
*Aeromonas hydrophila*	6	0.72%
*Staphylococcus haemolyticus*	6	0.72%
*Staphylococcus hominis*	6	0.72%
*Streptococcus pneumoniae*	6	0.72%
*Enterococcus gallinarum*	6	0.72%
*Streptococcus constellatus*	5	0.60%
*Proteus mirabilis*	5	0.60%
*Brucella melitensis*	5	0.60%
*Pseudomonas putida*	5	0.60%
*Serratia marcescens*	4	0.48%
*Streptococcus agalactiae*	3	0.36%
*Enterococcus lead*	3	0.36%
*Morganella morganii*	3	0.36%
*Micrococcus luteus*	2	0.24%
*Acinetobacter joni*	2	0.24%
*Streptococcus sanguinis*	1	0.12%
*Xanthomonas oryzae*	1	0.12%
*Citrobacter braque*	1	0.12%
*Fungi*	38	4.58%
**Total**	829	100%

### Analysis of drug sensitivity profiles of the top six pathogens isolated from patients with liver cirrhosis complicated by infection

The antimicrobial susceptibility results showed that *Escherichia coli* remained highly susceptible to imipenem, meropenem, amikacin, and tigecycline, with resistance rates of 1.08%, 1.08%, 1.08%, and 0%, respectively. In contrast, high resistance rates were observed for ampicillin (89.19%), cefazolin (63.24%), levofloxacin (55.14%), and trimethoprim–sulfamethoxazole (54.59%; Table 3). *Klebsiella pneumoniae* showed relatively low resistance to tobramycin (13.64%), amikacin (9.09%), and tigecycline (5.45%), but high resistance to ampicillin (100%), cefazolin (39.09%), and ampicillin–sulbactam (32.73%; Table 3). *Enterococcus faecium* was highly susceptible to linezolid, vancomycin, tigecycline, and quinupristin–dalfopristin, with resistance rates of 0% for all four agents. However, high resistance rates were observed for ampicillin, penicillin, ciprofloxacin, and levofloxacin, each at 93.42% (Table 3). *Acinetobacter baumannii* exhibited excellent susceptibility to tigecycline and polymyxin B, with no resistant isolates detected. Conversely, high resistance rates were observed for ampicillin–sulbactam (80.33%), piperacillin–tazobactam (81.97%), cefepime (78.69%), ceftazidime (78.69%), tobramycin (78.69%), amikacin (73.77%), and ciprofloxacin (55.74%; Table 3). *Staphylococcus aureus* showed excellent susceptibility to vancomycin, linezolid, quinupristin–dalfopristin, and moxifloxacin, with resistance rates of 0%, 1.89%, 0%, and 0%, respectively. However, high resistance rates were observed for penicillin (100%), erythromycin (67.92%), and oxacillin (32.08%; Table 3). According to CLSI M100 guidelines, susceptibility to piperacillin–tazobactam and other β-lactam agents in *S. aureus* is determined based on oxacillin susceptibility; therefore, oxacillin resistance serves as a surrogate marker for β-lactam non-susceptibility. *Pseudomonas aeruginosa* showed favorable susceptibility to cefepime, tobramycin, ciprofloxacin, and aztreonam, with resistance rates of 11.90%, 9.52%, 11.90%, and 11.90%, respectively. Moderate resistance rates were observed for imipenem (23.81%), meropenem (23.81%), piperacillin–tazobactam (19.05%), and cefoperazone–sulbactam (19.05%; Table 3). Notably, ampicillin, amoxicillin–clavulanic acid, penicillin, and erythromycin are not routinely tested or clinically interpretable for *P. aeruginosa*.

**Table 3:** Antimicrobial resistance profiles of the six leading pathogens isolated from cirrhotic patients with infection (2017–2025).

Antimicrobial agent	***Escherichia coli*** **(*****n*** = **185)**			***Klebsiella pneumoniae*** **(*****n*** = **110)**			***Enterococcus faecium*** **(*****n*** = **76)**			***Acinetobacter baumannii*** **(*****n*** = **61)**			***Staphylococcus aureus*** **(*****n*** = **53)**			***Pseudomonas aeruginosa*** **(*****n*** = **42)**		
	No. tested	No. resistant	Resistance rate (%)	No. tested	No. resistant	Resistance rate (%)	No. tested	No. resistant	Resistance rate (%)	No. tested	No. resistant	Resistance rate (%)	No. tested	No. resistant	Resistance rate (%)	No. tested	No. resistant	Resistance rate (%)
Ampicillin	185	165	89.19%	110	110	100.00%	76	71	93.42%	*NT*	*NT*	*NT*	*NT*	*NT*	*NT*	*NT*	*NT*	*NT*
Amoxicillin-clavulanic acid	185	58	31.35%	110	27	24.55%	*NT*	*NT*	*NT*	*NT*	*NT*	*NT*	*NT*	*NT*	*NT*	*NT*	*NT*	*NT*
Ampicillin-sulbactam	185	78	42.16%	110	36	32.73%	*NT*	*NT*	*NT*	61	49	80.33%	*NT*	*NT*	*NT*	*NT*	*NT*	*NT*
Piperacillin-tazobactam	185	13	7.03%	110	22	20.00%	*NT*	*NT*	*NT*	61	50	81.97%	*NT*	*NT*	*NT*	42	8	19.05%
Cefazolin	185	117	63.24%	110	43	39.09%	*NT*	*NT*	*NT*	*NT*	*NT*	*NT*	*NT*	*NT*	*NT*	*NT*	*NT*	*NT*
Cefepime	185	25	13.51%	110	22	20.00%	*NT*	*NT*	*NT*	61	48	78.69%	*NT*	*NT*	*NT*	42	5	11.90%
Ceftriaxone	185	94	50.81%	110	32	29.09%	*NT*	*NT*	*NT*	*NT*	*NT*	*NT*	*NT*	*NT*	*NT*	*NT*	*NT*	*NT*
Cefuroxime	185	100	54.05%	110	35	31.82%	*NT*	*NT*	*NT*	*NT*	*NT*	*NT*	*NT*	*NT*	*NT*	*NT*	*NT*	*NT*
Ceftazidime	185	34	18.38%	110	23	20.91%	*NT*	*NT*	*NT*	61	48	78.69%	*NT*	*NT*	*NT*	42	5	11.90%
Aztreonam	185	50	27.03%	110	28	25.45%	*NT*	*NT*	*NT*	*NT*	*NT*	*NT*	*NT*	*NT*	*NT*	42	5	11.90%
Imipenem	185	2	1.08%	110	9	8.18%	*NT*	*NT*	*NT*	61	35	57.38%	*NT*	*NT*	*NT*	42	10	23.81%
Meropenem	185	2	1.08%	110	9	8.18%	*NT*	*NT*	*NT*	61	32	52.46%	*NT*	*NT*	*NT*	42	10	23.81%
Gentamicin	185	58	31.35%	110	18	16.36%	*NT*	*NT*	*NT*	*NT*	*NT*	*NT*	53	6	11.32%	*NT*	*NT*	*NT*
Tobramycin	185	7	3.78%	110	15	13.64%	*NT*	*NT*	*NT*	61	48	78.69%	*NT*	*NT*	*NT*	42	4	9.52%
Amikacin	185	2	1.08%	110	10	9.09%	*NT*	*NT*	*NT*	61	45	73.77%	*NT*	*NT*	*NT*	*NT*	*NT*	*NT*
Ciprofloxacin	185	78	42.16%	110	35	31.82%	76	71	93.42%	61	34	55.74%	53	8	15.09%	42	5	11.90%
Levofloxacin	185	102	55.14%	110	32	29.09%	76	71	93.42%	61	25	40.98%	53	8	15.09%	42	5	11.90%
Tetracycline	185	56	30.27%	110	33	30.00%	76	37	48.68%	*NT*	*NT*	*NT*	53	16	30.19%	*NT*	*NT*	*NT*
Trimethoprim-sulfamethoxazole	185	101	54.59%	110	32	29.09%	*NT*	*NT*	*NT*	61	11	18.03%	53	1	1.89%	*NT*	*NT*	*NT*
Tigecycline	185	0	0.00%	110	6	5.45%	76	0	0.00%	61	0	0.00%	*NT*	*NT*	*NT*	*NT*	*NT*	*NT*
Ertapenem	185	2	1.08%	110	11	10.00%	*NT*	*NT*	*NT*	*NT*	*NT*	*NT*	*NT*	*NT*	*NT*	*NT*	*NT*	*NT*
Cefoperazone-sulbactam	185	2	1.08%	110	11	10.00%	*NT*	*NT*	*NT*	61	30	49.18%	*NT*	*NT*	*NT*	42	8	19.05%
Cefoxitin	185	29	15.68%	110	13	11.82%	*NT*	*NT*	*NT*	*NT*	*NT*	*NT*	*NT*	*NT*	*NT*	*NT*	*NT*	*NT*
Ticarcillin-clavulanic acid	*NT*	*NT*	*NT*	*NT*	*NT*	*NT*	*NT*	*NT*	*NT*	61	39	63.93%	*NT*	*NT*	*NT*	42	9	21.43%
Minocycline	*NT*	*NT*	*NT*	*NT*	*NT*	*NT*	*NT*	*NT*	*NT*	61	19	31.15%	*NT*	*NT*	*NT*	*NT*	*NT*	*NT*
Moxifloxacin	*NT*	*NT*	*NT*	*NT*	*NT*	*NT*	*NT*	*NT*	*NT*	*NT*	*NT*	*NT*	*NT*	*NT*	*NT*	*NT*	*NT*	*NT*
Polymyxin B	*NT*	*NT*	*NT*	*NT*	*NT*	*NT*	*NT*	*NT*	*NT*	61	0	0.00%	*NT*	*NT*	*NT*	42	0	0.00%
Penicillin	*NT*	*NT*	*NT*	*NT*	*NT*	*NT*	76	71	93.42%	*NT*	*NT*	*NT*	53	53	100.00%	*NT*	*NT*	*NT*
Vancomycin	*NT*	*NT*	*NT*	*NT*	*NT*	*NT*	76	0	0.00%	*NT*	*NT*	*NT*	53	0	0.00%	*NT*	*NT*	*NT*
Linezolid	*NT*	*NT*	*NT*	*NT*	*NT*	*NT*	76	0	0.00%	*NT*	*NT*	*NT*	53	1	1.89%	*NT*	*NT*	*NT*
Clindamycin	*NT*	*NT*	*NT*	*NT*	*NT*	*NT*	*NT*	*NT*	*NT*	*NT*	*NT*	*NT*	53	14	26.42%	*NT*	*NT*	*NT*
Quinupristin-dalfopristin	*NT*	*NT*	*NT*	*NT*	*NT*	*NT*	76	0	0.00%	*NT*	*NT*	*NT*	53	0	0.00%	*NT*	*NT*	*NT*
Rifampin	*NT*	*NT*	*NT*	*NT*	*NT*	*NT*	*NT*	*NT*	*NT*	*NT*	*NT*	*NT*	53	4	7.55%	*NT*	*NT*	*NT*
Oxacillin	*NT*	*NT*	*NT*	*NT*	*NT*	*NT*	*NT*	*NT*	*NT*	*NT*	*NT*	*NT*	53	17	32.08%	*NT*	*NT*	*NT*
Nitrofurantoin	*NT*	*NT*	*NT*	*NT*	*NT*	*NT*	76	40	52.63%	*NT*	*NT*	*NT*	*NT*	*NT*	*NT*	*NT*	*NT*	*NT*
Erythromycin	*NT*	*NT*	*NT*	*NT*	*NT*	*NT*	76	67	88.16%	*NT*	*NT*	*NT*	53	36	67.92%	*NT*	*NT*	*NT*

NT, not tested (antimicrobial agent not applicable for this organism per CLSI M100 guidelines, or not included in the testing panel); Resistance rates are reported as proportions of isolates tested (resistant strains/total tested). Nitrofurantoin results for E. faecium are reported from urinary isolates only (n = 76 from urine specimens).

### MDR and XDR isolates among cirrhotic patients with infections

Among the 791 bacterial isolates (excluding 38 fungal isolates), we identified 303 multidrug-resistant (MDR) isolates (38.3%) and 67 extensively drug-resistant (XDR) isolates (8.5%). Among the six leading pathogens, *Acinetobacter baumannii* exhibited the highest MDR rate (48/61, 78.7%), followed by *Escherichia coli* (144/185, 77.8%) and *Enterococcus faecium* (47/76, 61.8%). Regarding XDR isolates, *A. baumannii* again accounted for the largest proportion (27/61, 44.3%), followed by *K. pneumoniae* (17/110, 15.45%) and *E. coli* (23/185, 12.43%). No XDR isolates were detected among *Pseudomonas aeruginosa, E. faecium*, or *Staphylococcus aureus* (Table 4).

**Table 4:** The number of MDR and XDR isolates in cirrhotic patients with infections.

Organism	Total isolates	MDR isolates (*n*)	MDR rate (%)	XDR isolates (*n*)	XDR rate (%)
*Escherichia coli*	185	144	77.84%	23	12.43%
*Klebsiella pneumoniae*	110	42	38.18%	17	15.45%
*Acinetobacter baumannii*	61	48	78.70%	27	44.30%
*Pseudomonas aeruginosa*	42	5	11.90%	0	0.00%
*Enterococcus faecium*	76	47	61.84%	0	0.00%
*Staphylococcus aureus*	53	17	32.10%	0	0.00%

### Baseline characteristics of the gut microbiota subgroup

The demographic characteristics of the 86 participants enrolled in the gut microbiota subgroup are summarized in Table 5. No significant differences were observed among the HC, LCNOIF, and LCIF-SBP groups in age, sex distribution, or body mass index (all *P* > 0.05), indicating that the three groups were well matched at baseline. Table 6 further compares clinical and disease-severity characteristics between the LCNOIF and LCIF-SBP groups. Etiology of cirrhosis, presence of hepatic encephalopathy or gastrointestinal bleeding, comorbid diabetes mellitus and hypertension, and medication history (non-selective β-blockers, diuretics, lactulose, and proton pump inhibitors) did not differ significantly between the two groups (all *P* > 0.05). As expected, Child-Pugh class, Child-Pugh score, MELD score, and the presence of ascites were all significantly higher in the LCIF-SBP group (all *P* < 0.05). This finding is an inherent feature of the disease process under study rather than an independent confounder: spontaneous bacterial peritonitis occurs almost exclusively in patients with decompensated, ascitic cirrhosis (Biggins et al., 2021; European Association for the Study of the Liver, 2018), so greater disease severity in the LCIF-SBP group is expected and cannot be separated from SBP status in a cross-sectional comparison.

**Table 5:** Comparison of baseline demographic characteristics among the HC, LCNOIF, and LCIF-SBP groups (*n* = 86).

Characteristic	HC (*n* = 30)	LCNOIF (*n* = 30)	LCIF-SBP (*n* = 26)	*P*1	*P*2	*P*3
Age, years, mean ± SD	56.3 ± 9.7	53.1 ± 10.6	57.7 ± 11.6	0.224	0.618	0.123
Male, *n* (%)	18 (60.0%)	20 (66.7%)	15 (57.7%)	0.789	1.000	0.678
BMI, kg/m^2^, mean ± SD	22.6 ± 2.0	22.6 ± 2.8	22.2 ± 1.9	0.979	0.404	0.494

HC, healthy controls; LCNOIF, cirrhotic patients without infection; LCIF-SBP, cirrhotic patients with spontaneous bacterial peritonitis; BMI, body mass index.

P1, HC vs. LCNOIF; P2, HC vs. LCIF-SBP; P3, LCNOIF vs. LCIF-SBP. Overall comparisons across the three groups (one-way ANOVA for age and BMI, chi-square test for sex) were likewise non-significant (age, P = 0.244; sex, P = 0.769; BMI, P = 0.713), indicating that the three groups were well matched at baseline.

Continuous variables are presented as mean ± SD and were compared using the independent-samples t-test; categorical variables are presented as n (%) and were compared using the chi-square test.

**Table 6:** Clinical characteristics, disease severity, and medication history of the LCNOIF and LCIF-SBP subgroups.

Characteristic	LCNOIF (*n* = 30)	LCIF-SBP (*n* = 26)	*P*-value
Age, years, mean ± SD	53.1 ± 10.6	57.7 ± 11.6	0.123
Male, *n* (%)	20 (66.7%)	15 (57.7%)	0.678
BMI, kg/m^2^, mean ± SD	22.6 ± 2.8	22.2 ± 1.9	0.494
**Cirrhosis etiology**, ***n*** **(%)**			0.162
- Viral (HBV/HCV)	25 (83.3%)	16 (61.5%)	
- Alcoholic	2 (6.7%)	1 (3.8%)	
- Autoimmune	2 (6.7%)	5 (19.2%)	
- Other/unspecified	1 (3.3%)	4 (15.4%)	
**Child-Pugh class**, ***n*** **(%)**			<0.001
- Class A	26 (86.7%)	2 (7.7%)	
- Class B	4 (13.3%)	12 (46.2%)	
- Class C	0 (0.0%)	12 (46.2%)	
Child-Pugh score, median (IQR)	5 (5–6)	9 (7–11)	<0.001
MELD score, mean ± SD	11.0 ± 2.7	15.6 ± 6.0	0.002
Ascites, *n* (%)	1 (3.3%)	26 (100.0%)	<0.001
Hepatic encephalopathy, *n* (%)	0 (0.0%)	3 (11.5%)	0.188
Gastrointestinal bleeding, *n* (%)	1 (3.3%)	3 (11.5%)	0.504
Comorbidities, *n* (%)			
- Diabetes mellitus	7 (23.3%)	5 (19.2%)	0.963
- Hypertension	5 (16.7%)	4 (15.4%)	1.000
Medication history, *n* (%)			
- Non-selective β-blockers	8 (26.7%)	10 (38.5%)	0.512
- Diuretics (spironolactone ± furosemide)	9 (30.0%)	15 (57.7%)	0.069
- Lactulose	10 (33.3%)	13 (50.0%)	0.321
- Proton pump inhibitors	12 (40.0%)	14 (53.8%)	0.443
- Antibiotics or probiotics (past month)	0 (0.0%)	0 (0.0%)	–
Length of hospital stay, days, median (IQR)	10 (9–12)	12 (10–15)	0.024

LCNOIF, cirrhotic patients without infection; LCIF-SBP, cirrhotic patients with spontaneous bacterial peritonitis; BMI, body mass index; MELD, Model for End-Stage Liver Disease; IQR, interquartile range.

Continuous variables are presented as mean ± SD (compared by independent-samples t-test) or median (IQR) (compared by the Mann–Whitney U test) according to distribution; categorical variables are presented as n (%) and compared by the chi-square test.

All 26 LCIF-SBP participants met the diagnostic criteria for spontaneous bacterial peritonitis; ascites was therefore present in 100% of this group by definition, as SBP is an infection of pre-existing ascitic fluid, and Child-Pugh/MELD scores were correspondingly higher, reflecting that SBP occurs almost exclusively in decompensated cirrhosis.

Diuretic use was higher, but not significantly so, in the LCIF-SBP group; diuretics are frequently withheld during acute SBP because of the associated risk of acute kidney injury/hepatorenal syndrome, which narrows the between-group difference despite universal ascites in this group.

Length of hospital stay was modestly longer in the LCIF-SBP group, consistent with the additional course of intravenous antibiotic therapy required for SBP, but with substantial overlap between groups.

No participant in either group had received antibiotics or probiotics within the month preceding fecal sample collection, per the study exclusion criteria.

The length of hospital stay for the index admission was modestly longer in the LCIF-SBP group than in the LCNOIF group (*P* = 0.024), consistent with the additional course of intravenous antibiotic therapy required for SBP (Biggins et al., 2021; European Association for the Study of the Liver, 2018). Diuretic use did not differ significantly between the two groups (*P* = 0.069) despite universal ascites in the LCIF-SBP group, as diuretics are frequently withheld during acute SBP because of the associated risk of acute kidney injury and hepatorenal syndrome (Biggins et al., 2021). However, because fecal samples in both groups were collected within 24 h of admission and prior to the initiation of any in-hospital pharmacological treatment (as described in the Section Materials and methods), the total length of stay for that admission is a downstream, outcome-level variable relative to the sampling time point. It therefore could not have influenced the gut microbiota composition captured at baseline, and this between-group difference should not be interpreted as a pre-sampling confounder of the microbiota findings reported below.

### Analysis of α and β diversity of intestinal flora in patients with liver cirrhosis complicated with infection

**Figure 3:**
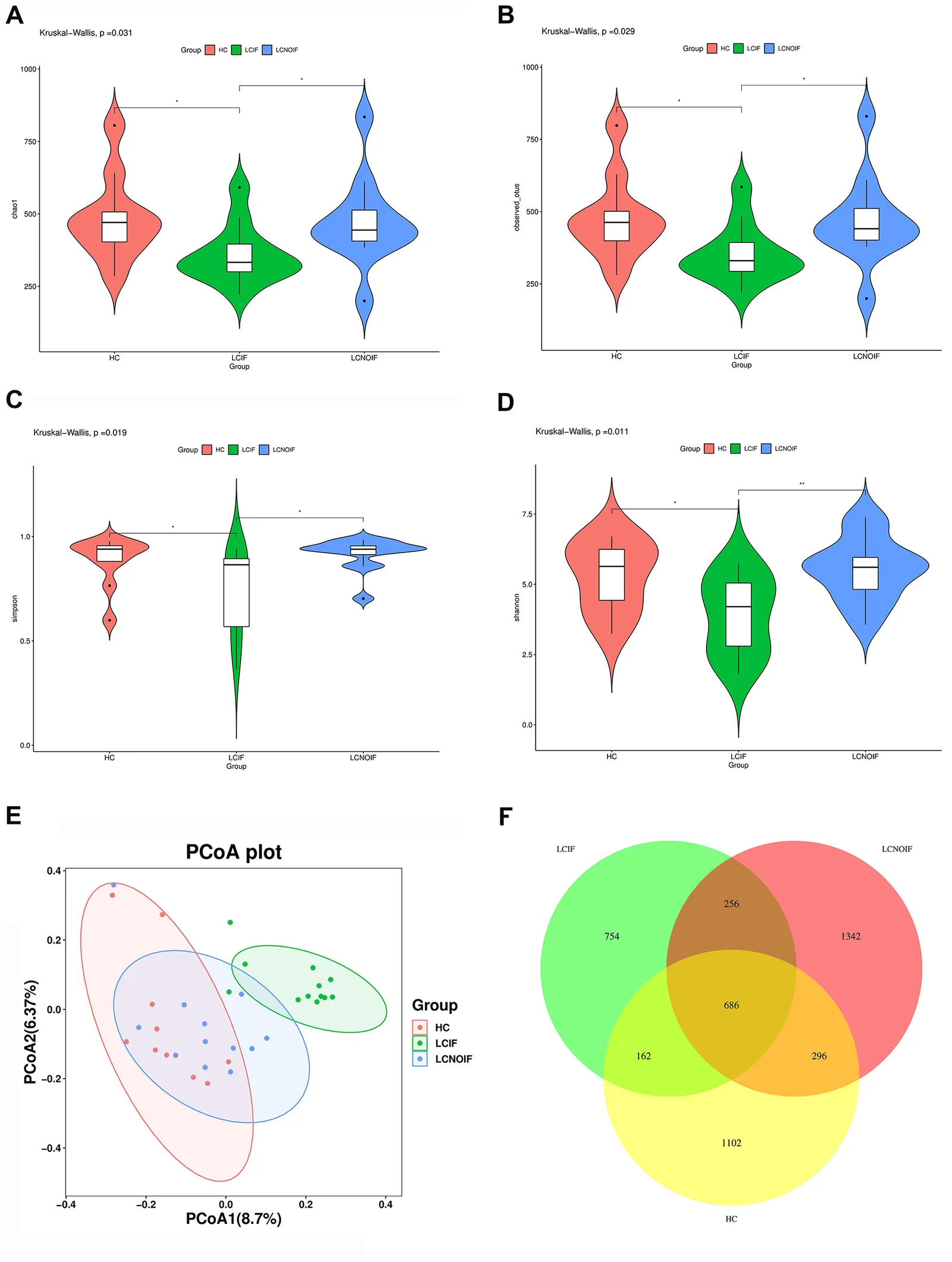
Alpha diversity, PCoA, and shared ASVs of gut microbiota among HC, LCNOIF, and LCIF-SBP groups. **(A–D)** Alpha diversity indices: Chao1, observed_ASVs, Simpson, and Shannon indices. **(E)** PCoA based on Bray-Curtis dissimilarity metrics. **(F)** Venn diagram displaying the number of unique and shared ASVs among the three groups. Venn diagram counts represent ASV-level features rather than formal species-level identifications. For clarity, “LCIF” in this figure refers to the LCIF-SBP subgroup.

To investigate gut microbial alterations specifically associated with peritoneal infection, we performed 16S rRNA sequencing on fecal samples from three groups: healthy controls (HC, *n* = 30), cirrhotic patients without infection (LCNOIF, *n* = 30), and cirrhotic patients with spontaneous bacterial peritonitis (LCIF-SBP, *n* = 26). The LCIF-SBP group represents a well-defined subset of the overall infected cohort, restricted to patients meeting the diagnostic criteria for SBP. Throughout this section, all microbiota-related analyses refer exclusively to the SBP subgroup. As illustrated in Figures 3A–D, significant differences in alpha diversity indices—including Chao1, observed_ASVs, Simpson, and Shannon—were observed between the HC and LCIF groups, as well as between the LCIF and LCNOIF groups. PCoA was employed to assess variations in community structure and community structure across samples (Figure 3E). The results indicated that the overall structure of the gut microbiota differed significantly between HC and LCIF, and between LCIF and LCNOIF (Figure 3E). Notably, the application of Venn diagrams in community analysis revealed that the HC, LCIF-SBP, and LCNOIF groups exhibited 1,913, 1,558, and 1,984 unique ASVs, respectively (Figure 3F). Meanwhile, the three groups shared a total of 686 core ASVs (Figure 3F).

### Cirrhosis complicated by infection altered the distribution of gut microbiota at the phylum and genus levels

**Figure 4:**
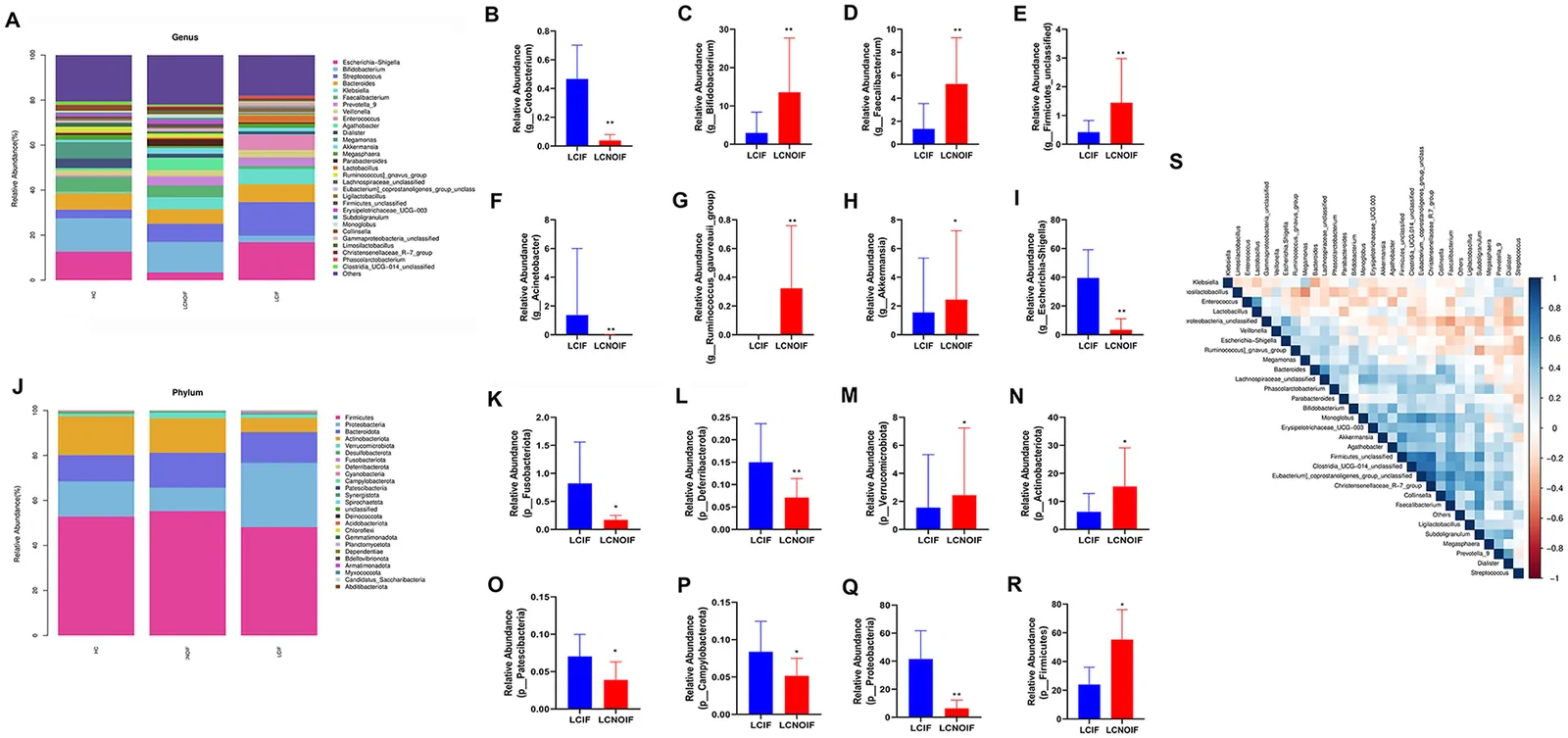
Alterations in gut microbiota composition at phylum and genus levels in LCIF-SBP infected cirrhotic patients. **(A–I)** Relative abundances of key bacterial genera showing significant differences among HC, LCNOIF, and LCIF-SBP groups. **(J–R)** Relative abundances of major bacterial phyla. **(S)** Correlation analysis of different bacterial genera. All taxonomic assignments represent genus-level classifications based on 16S rRNA V3–V4 sequencing and should not be interpreted as species-level identification. For clarity, “LCIF” in this figure refers to the LCIF-SBP subgroup.

To investigate the alterations in gut microbiota composition among patients with liver cirrhosis complicated by infection, we compared the relative abundance of microbial taxa across three groups: HC, LCIF, and LCNOIF (Figures 4A–R). Moreover, at the genus level, compared with LCNOIF, LCIF exhibited a significant decrease in the relative abundance of *Bifidobacterium, Faecalibacterium, Ruminococcus_gauvreauii*_*group, Firmicutes_unclassified*, and *Akkermansia*. Conversely, the relative abundance of *Cetobacterium, Escherichia/Shigella*, and *Acinetobacter* was significantly increased (Figures 4A–I). At the phylum level, the LCIF group exhibited significantly lower relative abundances of *Verrucomicrobiota, Actinobacteriota*, and *Firmicutes* compared to the LCNOIF group (Figures 4J–R). Conversely, the relative abundances of *Fusobacteria, Deferribacteres, Patescibacteria, Campylobacterota*, and *Proteobacteria* were markedly increased in the LCIF group (Figures 4J–R). Additionally, correlation analysis of different bacterial genera found that *Bifidobacterium* and *Faecalibacterium* exhibited negative correlations with *Escherichia/Shigella* (Figure 4S).

### Effects of liver cirrhosis combined with infection on short-chain fatty acids, metabolites of intestinal flora

**Figure 5:**
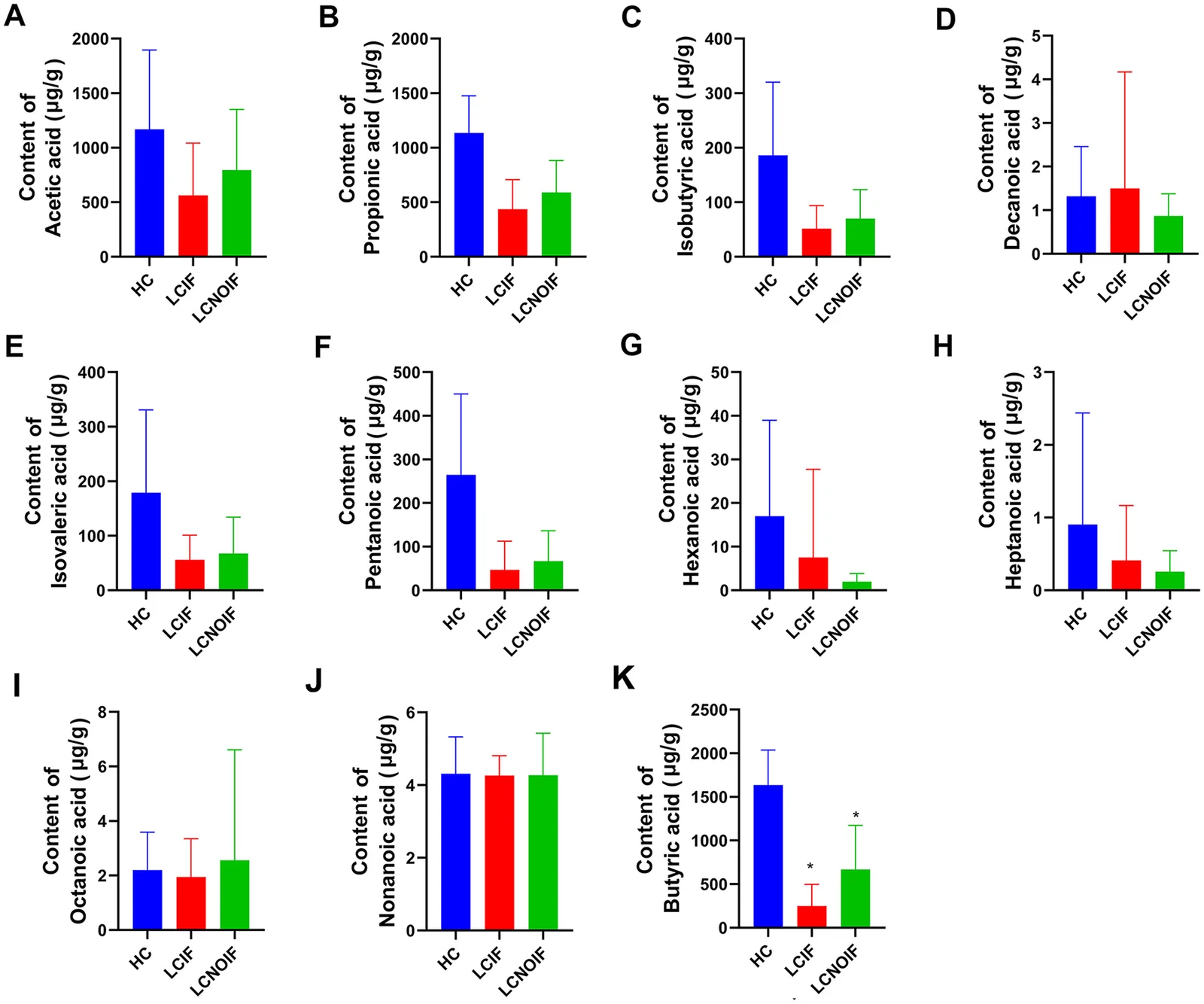
Short-chain fatty acid profiles and reduced butyrate levels in cirrhotic patients with infection. **(A–K)** Fecal concentrations of various short-chain fatty acids, including acetic acid, propionic acid, isobutyric acid, butyric acid, isovaleric acid, pentanoic acid, hexanoic acid, heptanoic acid, octanoic acid, nonanoic acid, and decanoic acid, across the three groups (HC, LCNOIF, and LCIF-SBP). For clarity, “LCIF” in this figure refers to the LCIF-SBP subgroup.

The metabolite subdivisions of fecal samples from patients in each group are shown in Figures 5A–K. Meanwhile, the results indicated that the levels of butyrate, a metabolite of gut microbiota, were significantly lower in cirrhotic patients without infection compared to healthy controls (Figure 5K). Furthermore, butyrate levels were even more markedly reduced in cirrhotic patients with concomitant infection when compared to those without infection (Figure 5K).

### Correlation analysis of gut microbiota, short-chain fatty acids, and laboratory parameters in SBP patients

**Figure 6:**
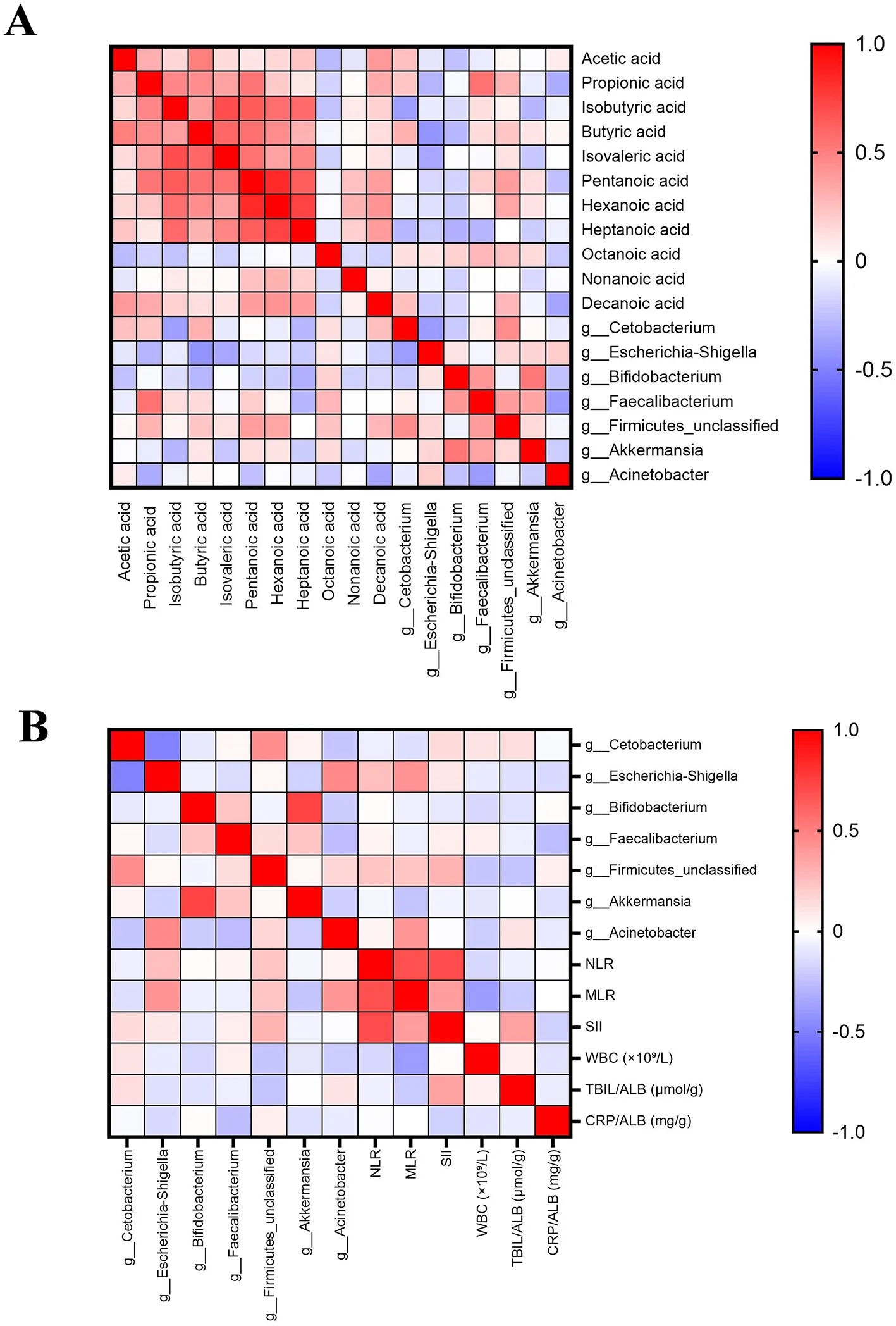
Spearman correlation analysis of gut microbiota, short-chain fatty acids (SCFAs), and laboratory inflammatory parameters in patients with spontaneous bacterial peritonitis (LCIF-SBP). **(A)** Heatmap showing Spearman correlation coefficients between differentially abundant bacterial genera, fecal SCFA levels, and key laboratory inflammatory indices in cirrhotic patients with SBP. **(B)** Correlation matrix among clinical laboratory parameters (NLR, MLR, and SII). Color intensity represents correlation strength (red: positive; blue: negative). All taxonomic assignments represent genus-level classifications based on 16S rRNA V3–V4 sequencing and should not be interpreted as species-level identification.

To further explore the interrelationships among gut microbiota, intestinal metabolites, and host inflammatory status, we performed Spearman correlation analysis incorporating the most differentially abundant bacterial genera, fecal SCFA levels, and key laboratory indices in patients with liver cirrhosis and spontaneous bacterial peritonitis (Figure 6).

In terms of inter-bacterial associations, Bifidobacterium showed a strong positive correlation with Akkermansia (*r* = 0.735, *P* < 0.001), suggesting that these two SCFA-producing genera may co-inhabit and mutually reinforce a health-associated gut environment. Additionally, *Escherichia/Shigella* was positively associated with Acinetobacter (*r* = 0.461, *P* = 0.018), implying that potentially pathogenic genera may co-expand in the context of infection-associated dysbiosis (Figure 6A).

With respect to the relationship between gut microbiota and fecal SCFAs, *Escherichia/Shigella* demonstrated a significant negative association with butyric acid levels (*P* = 0.040), while *Faecalibacterium* showed a significant positive association with propionic acid (*P* = 0.005; Figure 6A). Given the exploratory nature of these analyses and the modest sample size, these correlations should be interpreted as hypothesis-generating rather than confirmatory, these findings imply that alterations in SCFA-producing microbiota may contribute to the disruption of mucosal homeostasis in SBP patients.

Regarding the associations between gut microbiota and laboratory inflammatory indices, *Escherichia/Shigella* and *Acinetobacter* were both significantly positively correlated with the MLR (*r* = 0.427, *P* = 0.029 and *r* = 0.414, *P* = 0.035, respectively), suggesting that the expansion of these genera that include clinically relevant species may be linked to monocyte-mediated systemic inflammatory activation. Among the clinical laboratory parameters, NLR showed strong positive correlations with both MLR (*r* = 0.683, *P* < 0.001) and SII (*r* = 0.702, *P* < 0.001; Figure 6B). These findings indicate that the overgrowth of genera that include clinically relevant species was positively correlated with systemic inflammatory markers, whereas the depletion of SCFA-producing genera was negatively correlated with fecal SCFA levels. These correlation patterns suggest a potential association between gut microbial dysbiosis and disease progression, although causal inference cannot be established due to the cross-sectional design.

## Discussion

Infection poses a significant burden as a major complication in patients with cirrhosis. The early identification and effective treatment of infections are key clinical priorities in managing cirrhosis, as they are associated with improved patient outcomes.

This study focused on comparing the laboratory test indexes of LCIF patients and LCNOIF patients. The results showed that there were significant differences in several non-invasive laboratory test indexes between the two groups, and ROC curve analysis further confirmed the diagnostic utility of these indicators in identifying infection among cirrhotic patients. NLR is an important indicator of inflammation and immune status. Some studies have pointed out that NLR has important value in the diagnosis of infectious diseases (Agarwal, 2022). In the present study, NLR achieved an AUC of 0.9447, demonstrating excellent discriminatory performance between the LCIF and LCNOIF groups. The MLR can also reflect the inflammatory and immune balance of the body. Relevant literature shows that MLR has a certain application prospect in the diagnosis of infection (Bothamley, 2024), a finding corroborated by our results, in which MLR yielded an AUC of 0.9083. The SII integrates the information of platelets, neutrophils and lymphocytes, which can more comprehensively reflect the immune inflammation state of the body (Wang et al., 2023). In our cohort, SII demonstrated good diagnostic performance with an AUC of 0.8462. White blood cell count is one of the commonly used clinical infection detection indicators. Previous studies have shown that white blood cell count has a certain specificity and sensitivity in the diagnosis of infectious diseases (Liao et al., 2021). The TBIL/ALB ratio reflects the metabolic and synthetic functions of the liver and the nutritional status of the body. Studies have shown that TBIL/ALB can be used as an indicator to evaluate the severity and prognosis of the disease in infectious diseases (Yang et al., 2025). However, in the present study, the ROC curve for TBIL/ALB approached the reference diagonal with an AUC of only 0.5919, indicating that despite being significantly elevated in the LCIF-SBP group, TBIL/ALB has limited standalone discriminatory ability for infection diagnosis and may be more reflective of underlying hepatic impairment than of active infection *per se*. Although ALT/AST was found to be lower in LCIF patients than in LCNOIF patients in this study, the difference was not statistically significant. However, studies have shown that ALT/AST can also change in some infectious diseases (Liu et al., 2025). Therefore, NLR, MLR, and SII demonstrated significant associations with infection status in this cohort and may serve as exploratory adjunctive indicators to raise clinical suspicion of whether patients with liver cirrhosis are complicated with infection, with NLR, and MLR showing the strongest diagnostic performance. These indicators can be obtained by routine blood tests, which are simple to operate and low cost, and suitable for wide clinical application.

Patients with liver cirrhosis frequently face challenges posed by MDR bacteria, a problem that has become increasingly prominent in clinical practice. MDR infections not only increase mortality rates but also raise the bar for treatment options. Studies show that cirrhosis patients experience higher infection rates, particularly hospital-acquired (HCAi) and community-acquired (CAi) infections (Grgurevic et al., 2020). Notably, MDR bacteria are significantly more prevalent in these infections, especially in hospital settings, which further exacerbates liver damage and mortality risks (Grgurevic et al., 2020). Varghese et al. (Varghese et al., 2025) observed an even higher MDR rate (54%) among cirrhotic patients in northern India, with carbapenem-resistant organisms comprising 22.6% of Gram-negative isolates. In contrast, our cohort showed a relatively lower MDR burden but similarly high susceptibility to carbapenems, suggesting that resistance profiles vary considerably across geographic regions within Asia. Our investigation revealed that in patients with cirrhosis complicated by infections, Gram-negative bacteria predominate the infection spectrum, with *Escherichia coli, Klebsiella pneumoniae, Acinetobacter baumannii*, and *Pseudomonas aeruginosa* being the most prevalent pathogens. This observation is consistent with existing literature. Research indicates that Gram-negative bacteria are the primary pathogens responsible for SBP in cirrhotic patients. One study on SBP in this patient population demonstrated that Gram-negative bacteria constitute a significant proportion of these infections, with *Escherichia coli* being particularly common (Mittal et al., 2020). Furthermore, another study emphasized that Gram-negative bacteria remain the principal source of infection in patients with acute decompensated cirrhosis, and the emergence of multidrug-resistant strains poses additional challenges for treatment (Liu et al., 2020). Additionally, evidence suggests that Klebsiella pneumoniae is a frequent pathogen among cirrhotic patients with bacterial pneumonia, exhibiting multidrug resistance to various antibiotics, thereby complicating therapeutic interventions (Zhang et al., 2020). Concurrently, *Acinetobacter baumannii* has been consistently identified as a multidrug-resistant bacterium across multiple studies, with its high incidence among cirrhosis patients highlighting its significance in clinical management (Grgurevic et al., 2020). Additionally, *Pseudomonas aeruginosa*, another common Gram-negative bacterium, exhibits a notable infection rate in cirrhosis patients. Studies reveal that infections caused by *Pseudomonas aeruginosa* are not only widespread but also present significant treatment challenges due to its drug resistance (Schultze et al., 2021). Moreover, the immunocompromised condition and extended hospital stays associated with cirrhosis patients further elevate the risk of *Pseudomonas aeruginosa* infection (Hsu et al., 2021).

In addition, our study revealed that various pathogens exhibit distinct drug resistance profiles. For instance, *Escherichia coli* demonstrated excellent susceptibility to imipenem, meropenem, amikacin, and tigecycline (resistance rates of 1.08%, 1.08%, 1.08%, and 0%, respectively), while exhibiting high resistance rates to ampicillin (89.19%), cefazolin (63.24%), levofloxacin (55.14%), and trimethoprim-sulfamethoxazole (54.59%; Table 3). Conversely, *Pseudomonas aeruginosa* showed favorable susceptibility to cefepime, tobramycin, and ciprofloxacin (resistance rates of 11.90%, 9.52%, and 11.90%, respectively), but exhibited relatively higher resistance to imipenem and meropenem (both 23.81%; Table 3). It should be noted that for *Staphylococcus aureus*, CLSI M100 guidelines recommend that susceptibility to piperacillin-tazobactam be determined by oxacillin susceptibility. Therefore, the high oxacillin resistance rate (32.08%) observed in our cohort serves as a surrogate indicator of piperacillin-tazobactam non-susceptibility, consistent with standard microbiological practice. These results highlight the critical importance of judicious antibiotic use in managing infections among patients with liver cirrhosis. Empirical antibiotic therapy should be informed by local pathogen prevalence and resistance patterns and subsequently refined based on pathogen identification and drug sensitivity testing outcomes.

The analysis of gut microbiota diversity using 16S rRNA sequencing revealed significant alterations in the intestinal flora of patients with spontaneous bacterial peritonitis compared to both healthy controls and cirrhotic patients without infection. Alpha diversity indices, including Chao1, observed Otus, Simpson, and Shannon, were significantly lower in the LCIF-SBP group compared to the HC and LCNOIF groups. This reduction in alpha diversity indicates a loss of microbial richness and evenness, which is often associated with impaired gut barrier function and increased susceptibility to infections (Leibovitzh et al., 2022). PCoA further demonstrated that the overall structure of the gut microbiota differed significantly between the three groups. UPGMA confirmed that the microbiota composition was distinctly clustered among the HC, LCIF-SBP, and LCNOIF groups. These findings suggest that the presence of infection in cirrhotic patients leads to a distinct shift in the gut microbial community, potentially contributing to disease progression and poor outcomes.

At the phylum level, the LCIF-SBP group demonstrated significantly reduced relative abundances of *Verrucomicrobiota* and *Actinobacteriota* in comparison to the LCNOIF group. *Verrucomicrobiota*, particularly *Akkermansia muciniphila*, are recognized for their critical role in maintaining gut barrier integrity through the degradation of mucin (Wang et al., 2024; Zhao et al., 2024). Consequently, the observed reduction in *Verrucomicrobiota* among infected cirrhotic patients may contribute to enhanced intestinal permeability and bacterial translocation, which are important factors in the pathogenesis of sepsis and systemic inflammation (Xie et al., 2023; Cani et al., 2022). Conversely, the relative abundances of *Proteobacteria, Fusobacteria, Deferribacteres, Patescibacteria*, and *Campylobacterota* were markedly increased in the LCIF-SBP group. These taxa are frequently associated with inflammatory conditions and may support immune dysregulation in cirrhotic patients (Hong et al., 2023; Li et al., 2021; Liu B. et al., 2022; He et al., 2023). For instance, *Fusobacterium* species have been implicated in the exacerbation of inflammatory bowel disease and may contribute to the pathogenesis of liver-related infections (Quaglio et al., 2022; Buelow et al., 2013; Chauhan and Gidwani, 2020).

At the genus level, the LCIF-SBP group exhibited a significant reduction in the relative abundance of *Bifidobacterium, Faecalibacterium, Agathobacter, Akkermansia*, the *Ruminococcus gnavus* group, and unclassified *Firmicutes* compared to the LCNOIF group. These genera are recognized for their functional roles in gut health, including the production of SCFAs and the maintenance of immune homeostasis (Turroni et al., 2019; Xiao et al., 2025; Li et al., 2025; Liu M. J. et al., 2022; Lukovac et al., 2014; Ferreira-Halder et al., 2017). The depletion of these commensal bacteria may compromise the host's ability to regulate inflammation and resist infection, potentially exacerbating the clinical progression of cirrhosis. Conversely, the relative abundance of *Escherichia/Shigella, Acinetobacter*, and *Cetobacterium* was significantly elevated in the LCIF-SBP group. Although some of these genera are components of the normal gut microbiota, their overrepresentation in infected cirrhotic patients may indicate a dysbiosis favoring genera that include clinically relevant species (García-Solache and Rice, 2019; Amorim and Nascimento, 2017; Qi et al., 2023). In the context of liver cirrhosis, *Escherichia/Shigella* infections often lead to decompensation in cirrhotic patients, increasing the risk of acute-on-chronic liver failure (ACLF) and other complications (Cannon et al., 2020; Rodina et al., 2021). Moreover, *Escherichia/Shigella* are associated with antibiotic resistance and nosocomial infections, which could further complicate the management of cirrhotic patients (Miller and Arias, 2024; Denissen et al., 2022). Correlation analysis revealed negative associations between *Bifidobacterium, Faecalibacterium*, and *Escherichia/Shigella*. This suggests that the expansion of genera that include potentially pathogenic species may come at the expense of SCFA-producing commensals, leading to a state of dysbiosis that could exacerbate liver damage and increase the risk of infection.

SCFAs are key metabolites produced by the intestinal microbial community and play diverse physiological roles, including the regulation of intestinal barrier function, immune responses, and energy metabolism (Zhang et al., 2023; Mann et al., 2024). The current study identified significantly reduced butyrate levels in patients with cirrhosis, irrespective of co-infection status, compared to healthy controls. Furthermore, butyrate levels were even more diminished in patients with both cirrhosis and co-infection. These findings imply that cirrhosis alone may adversely impact the metabolic function of the gut microbiota, with co-infection further exacerbating this effect. Butyrate serves as the primary energy substrate for intestinal epithelial cells, and its deficiency can result in compromised intestinal barrier function, consequently elevating the risk of infection (Salvi and Cowles, 2021). Furthermore, butyrate exhibits anti-inflammatory properties, capable of suppressing the expression of pro-inflammatory mediators and attenuating the intestinal inflammatory response (Souders et al., 2023; Ferrer-Picón et al., 2020). Consequently, the marked reduction in butyrate levels observed in cirrhotic patients with infections may be associated with intestinal barrier dysfunction and an exacerbated inflammatory response.

The correlation patterns observed in this study provide further mechanistic insights into the interplay between gut dysbiosis, SCFA metabolism, and systemic inflammation in SBP patients. The strong positive correlation between *Bifidobacterium* and *Akkermansia* (*r* = 0.735, *P* < 0.001) is consistent with their shared role in maintaining mucosal barrier integrity and modulating host immune homeostasis; depletion of either genus in the context of cirrhosis-related infection likely amplifies intestinal permeability and supports bacterial translocation (Mo et al., 2024). The concurrent positive association between *Escherichia-Shigella* and *Acinetobacter* (*r* = 0.461, *P* = 0.018) suggests that these genera that include clinically relevant species may co-expand under infection-associated dysbiosis, consistent with prior reports demonstrating that dominance of *Escherichia/Shigella* in decompensated cirrhosis correlates with reduced SCFA levels and activation of pathogenicity-related metabolic pathways (Baltazar-Díaz et al., 2022). The negative association between *Escherichia-Shigella* and butyric acid, together with the positive association between *Faecalibacterium* and propionic acid, highlights a functional consequence of microbiota remodeling in SBP. Butyrate, primarily produced by *Faecalibacterium prausnitzii*, exerts potent anti-inflammatory effects via HDAC inhibition and suppression of pro-inflammatory signaling, and its deficiency compromises epithelial barrier integrity (Zhang et al., 2019). Furthermore, the significant positive correlations of *Escherichia-Shigella* and *Acinetobacter* with MLR suggest that the expansion of these pathobionts may drive monocyte-mediated inflammatory amplification, a notion supported by evidence that elevated MLR reflects a pro-inflammatory immune microenvironment and is associated with poor outcomes in liver-related disease (Wang et al., 2022). The strong inter-correlations among NLR, MLR, and SII further reflect the multidimensional nature of immune dysregulation in this patient population (Yang et al., 2022).

In conclusion, this study conducted a systematic analysis of pathogen distribution, drug resistance characteristics, alterations in the intestinal microbial community, and SCFA metabolism in patients with liver cirrhosis and infection in Lanzhou. The findings indicated that Gram-negative bacteria were predominantly present in patients with liver cirrhosis complicated by infection, accompanied by significant issues of drug resistance. There was a marked reduction in the diversity of the intestinal microbial community, characterized by a decrease in SCFA-producing genera and an increase in genera that include clinically relevant pathogens (e.g., *Escherichia/Shigella*). Concurrently, butyrate levels were significantly diminished, suggesting that an imbalance in the intestinal microecology may play a critical role in the onset and progression of liver cirrhosis complicated by infection.

Furthermore, the increased abundance of potential genera that include clinically relevant species, such as *Escherichia/Shigella, Acinetobacter*, and *Pseudomonas* in the fecal microbiota of the LCIF-SBP group was observed. These genera include species such as *Escherichia coli*, and *Acinetobacter baumannii*, which are frequently detected among bacterial pathogens in patients with liver cirrhosis and infections. This comparison operates at the genus-to-species level and should be interpreted as population-level concordance rather than individual-level identification. This finding suggests that fecal microbiota analysis may be indirectly associated with the detection of bacterial infections in liver cirrhosis patients. Therefore, the analysis of gut microbiota in fecal samples may provide complementary, non-invasive information associated with infection status in this patient population and may serve as a potential adjunctive biomarker for assessing bacterial infections in this region. However, further prospective, large-scale, and externally validation are required before this approach can be considered for routine clinical application.

Several limitations of this study should be acknowledged. First, the retrospective design of the clinical data collection may have introduced selection bias and incomplete record-keeping, despite the use of a standardized hospital information system. Second, this was a single-center study conducted in Lanzhou, northwestern China; therefore, the pathogen distribution and antimicrobial resistance patterns observed may not be generalizable to other geographic regions with different epidemiological profiles and antibiotic stewardship practices. While our findings provide valuable region-specific data for Lanzhou and its surrounding areas, they may not fully represent the broader Northwest China region or other healthcare settings. Future multicenter studies incorporating data from multiple hospitals across Northwest China are warranted to validate and extend our findings. Third, the sample size for the gut microbiota and metabolomics analyses (*n* = 86) was relatively modest, and the microbiota subgroup was restricted to patients with spontaneous bacterial peritonitis, which limits the extrapolation of these findings to other types of infections in cirrhotic patients. Furthermore, the 16S rRNA gene sequencing employed in this study provides taxonomic resolution primarily at the genus level. Therefore, the Venn diagram counts presented in Figure 3F represent ASV-level shared and unique features rather than formal species-level identifications. Species-level assignments should be interpreted with caution, as closely related species within the same genus may share identical or nearly identical V3–V4 sequences. Metagenomic sequencing approaches, which offer species-level resolution and enable simultaneous analysis of antimicrobial resistance genes (ARGs) and functional pathways, could provide deeper insights in future investigations. Fourth, although we collected fecal samples prior to antibiotic administration to minimize confounding, we did not account for potential confounders such as dietary habits, proton pump inhibitor use, or detailed medication histories, which are known to influence gut microbiota composition. Importantly, the cross-sectional design of the microbiota and metabolomics analyses precludes any causal inference. We cannot definitively determine whether the observed gut dysbiosis and reduced butyrate levels represent a predisposing factor that promotes bacterial translocation and subsequent infection, or whether they are a consequence of the acute inflammatory response to infection itself or reflect the combined effects of multiple confounding factors. Fifth, we did not perform longitudinal follow-up to evaluate the dynamic changes in gut microbiota or SCFA levels during infection resolution or disease progression. Fifth, WBC and CRP/ALB were excluded from the ROC analysis in this study because both parameters were components of the clinical criteria used to define infection status, which could introduce incorporation bias and inflate the observed AUC values. The ROC analyses for the remaining indicators should therefore be considered exploratory, and external validation in an independent cohort is warranted before these indicators can be recommended as standalone diagnostic tools. Notably, NLR, MLR, and SII are derived composite indices not directly included in the infection diagnostic criteria, which may partially mitigate this concern for these specific parameters. We addressed major dietary and pharmacological confounders in this study through strict exclusion criteria applied to the microbiota subgroup—excluding patients with recent antibiotic or probiotic use, special dietary regimens, and comorbidities known to influence gut microbiota—and by recording and comparing baseline use of proton pump inhibitors and lactulose between the LCNOIF and LCIF-SBP groups, which did not differ significantly (Table 6). Nevertheless, more granular data—such as habitual dietary intake and detailed medication-exposure histories—were not systematically collected. Future studies incorporating these elements would further strengthen causal inference in this area. Finally, the antimicrobial susceptibility testing was based on phenotypic methods without molecular characterization of resistance genes, which precludes a better understanding of the underlying resistance mechanisms. Future multicenter prospective studies with larger sample sizes and integrated metagenomic and metabolomic approaches are warranted to validate and extend our findings. In addition, mechanistic validation through animal models of cirrhosis, fecal microbiota transplantation (FMT), and butyrate supplementation experiments would help determine whether the observed gut dysbiosis and reduced butyrate levels are a cause or consequence of infection, and whether restoring microbial homeostasis can improve clinical outcomes in cirrhotic patients.

Collectively, our findings provide integrated clinical and microbiological evidence that gut microbial dysbiosis and altered microbial metabolism are closely associated with infection in patients with liver cirrhosis. These observations suggest that gut microbiota composition and metabolite profiles are associated with infection status in cirrhotic patients and may offer insights for future microbiota-targeted research. However, whether the observed gut dysbiosis represents a predisposing factor, a consequence of infection, or a marker of disease progression and antibiotic exposure remains unresolved due to the observational and cross-sectional nature of this study. Future longitudinal multicenter studies with serial sampling, together with mechanistic investigations using animal models and fecal microbiota transplantation, as well as shotgun metagenomic sequencing, will be essential to clarify the causal relationship and further elucidate the underlying mechanisms.

## Data Availability

The 16S rRNA gene sequencing data generated in this study have been deposited in the NCBI BioProject database under accession number PRJNA1401534 (https://www.ncbi.nlm.nih.gov/bioproject/PRJNA1401534). The clinical datasets analyzed during the current study are not publicly available due to patient privacy restrictions but are available from the corresponding author upon reasonable request.
